# Personalized medicine in diabetes: the role of ‘omics’ and biomarkers

**DOI:** 10.1111/dme.13075

**Published:** 2016-05-19

**Authors:** E. R. Pearson

**Affiliations:** ^1^ Division of Cardiovascular and Diabetes MedicineMedical Research InstituteUniversity of DundeeDundeeUK

## Abstract

Personalized medicine, otherwise called stratified or precision medicine, aims to better target intervention to the individual to maximize benefit and minimize harm. This review discusses how diabetes aetiology, pathophysiology and patient genotype influence response to or side effects of the commonly used diabetes treatments. C‐peptide is a useful biomarker that is underused to guide treatment choice, severe insulin deficiency predicts non‐response to glucagon‐like peptide‐1 receptor agonists, and thiazolidinediones are more effective in insulin‐resistant patients. The field of pharmacogenetics is now yielding clinically important results, with three examples outlined: sulphonylurea sensitivity in patients with HNF1A maturity‐onset diabetes of the young; sulphonylurea sensitivity in patients with Type 2 diabetes with reduced function alleles at CYP2C9, resulting in reduced metabolism of sulphonylureas; and severe metformin intolerance associated with reduced function organic cation transporter 1 (OCT1) variants, exacerbated by drugs that also inhibit OCT1. Genome‐wide approaches and the potential of other ‘omics’, including metagenomics and metabolomics, are then outlined, highlighting the complex interacting networks that we need to understand before we can truly personalize diabetes treatments.

## Personalized medicine: from art to science

The practice of clinical medicine teaches us to assess each patient and, on the basis of their symptoms, signs and targeted investigations, to develop a personalized management plan. When we manage patients with diabetes, it is clear that they represent a very diverse group of people, spanning all ethnicities, the young to the old, the slim to the morbidly obese, the insulin‐deficient to the markedly insulin‐resistant. As clinicians we try to take into account these differences when developing a personalized management plan with our patients. This process of personalizing therapy currently is often more of an art than a science.

The joint American Diabetes Association/European Association for the Study of Diabetes position statement for the management of hyperglycaemia in Type 2 diabetes 1 does move guidelines away from a step‐by‐step protocol‐driven approach and encourages us to consider a patient‐centred approach. In this position statement the efficacy and side effects of each diabetes drug class are presented with a recommendation that ‘choice is based on patient preferences as well as various patient, disease, and drug characteristics, with the goal being to reduce glucose concentrations while minimizing side effects, especially hypoglycaemia’. This approach is sensible, pragmatic and largely based on common sense, e.g. avoiding sulphonylureas in those who are vulnerable to hypoglycaemia, or where hypoglycaemia would be of considerable risk such as in lorry drivers or scaffolders. Yet whilst common sense would suggest to avoid a weight‐gaining therapy in someone who is obese, thiazolidinediones appear to be more effective in insulin‐resistant individuals; how much should this improvement in HbA_1c_ be balanced against the increased weight gain? We need evidence to guide these decisions, which requires trials specifically aimed to assess what drug is ‘best’ for an individual.

In addition to phenotypic heterogeneity of patients with diabetes, we see diversity in response to treatment or outcome of disease, despite similarity in phenotype: why does one person end up requiring insulin treatment within 3 years of diagnosis, and another phenotypically similar person not progress to insulin for > 15 years? Why does one person develop diabetic retinopathy and another not, despite both having 20 years of good glycaemic control? Heritability studies are useful here, as they tell us how much of the variability between individuals can be explained by genetic differences. The FIND‐eye study 2 reported a broad sense heritability for diabetic retinopathy of ~27% and we have recently reported heritability for glycaemic response to metformin at ~34% 3. Thus, a considerable percentage of variability in patient response or outcome is ‘intrinsic’ to that individual, and this may well not be apparent in their phenotype.

For a truly personalized approach to management of patients of diabetes we need: 1) to better understand how clinical phenotypic variation alters response or outcome; 2) to identify molecular signatures (‘omics’) that improve our ability to predict outcome; and 3) to establish that knowing 1 and 2 will lead to a change in patient management and improved patient care and outcome. In this way we should be able to capture at least some of the ‘art of medicine’ and provide a scientific rationale and evidence for personalized care.

## To personalize, stratify or be precise?

**Figure 1:**
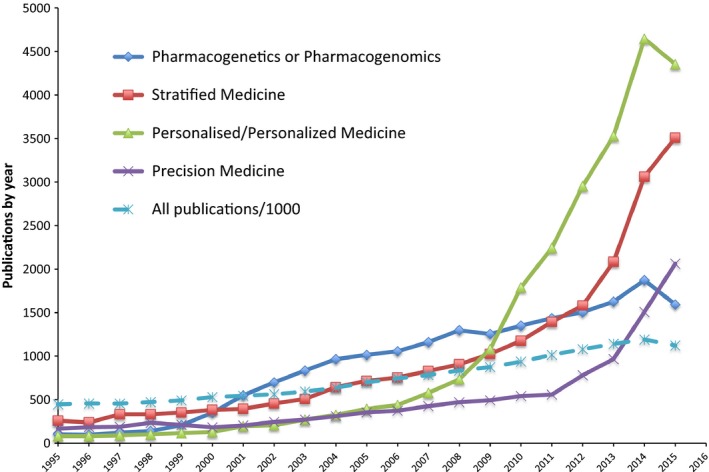
The number of publications per year where the search term was in the title. The search terms were (Pharmacogenetics OR Pharmacogenomics), ‘Stratified Medicine’, (‘Personalised Medicine’ OR ‘Personalized Medicine’), ‘Precision Medicine’. All publications (dashed line) were restricted by year with no search term and the total number was divided by 1000 to enable use of the same scale.

The field of personalized medicine is an area of ever‐changing terminology (Fig. 1). In 1995–2005, the ability to personalize treatment was largely considered the realm of pharmacogenetics, or pharmacogenomics (a term used to express studies across the whole genome). After a surge in pharmacogenetic/‐omic studies during this time, the publication rate of articles in this area has largely increased in line with the background population of published papers. The concept of personalized medicine really took off during 2007/2008 and remains a popular term; however, as it became apparent that it would be hard to truly individualize or personalize treatment, the term ‘stratified medicine’ became popular, the concept being that subgroups or strata of individuals should be treated differently from other strata. The final twist came with the concept of ‘precision medicine’, which describes the use of clinical and ‘omic’ characteristics to enable a more precise treatment, i.e. one that is more accurate, with less error (or fewer side effects). This term was slowly emerging before this year, but the launch of the Precision Medicine Initiative in the USA by President Obama in his state of the nations address in January 2015 has made this a highly trending term in the literature.

When considering all these terms, it is apparent that the field of personalized/precision medicine is dominated at present by cancer therapies, where there is the unique ability to obtain tissue from the target tissue and to identify somatic mutations that will enable therapy that only acts on the cancer. In the last 10 years, 12 times more cancer studies have been published than diabetes studies in this area. In the present review, I will use the term ‘personalized’, and highlight key developments in personalized medicine in diabetes from the last 10 years, and how the field continues to evolve, especially in molecular or ‘omic’ space. I will focus on glycaemia in non‐Type 1 diabetes, rather than other aspects of care, and in particular on glycaemic response to therapies.

## Diabetes pathophysiology

In patients with Type 2 diabetes, for a given level of glycaemia, some patients will have marked insulin resistance, with robust but insufficient insulin secretion, while others will have very low insulin secretion but be very, but insufficiently, insulin‐sensitive. Given that the diabetes treatments work to promote insulin secretion (sulphonylureas, dipeptidyl peptidase‐4 inhibitors, glucagon‐like peptide‐1 receptor agonists) or to promote insulin action (thiazolidinediones) or independently of the insulin secretion/sensitivity axis (sodium‐glucose cotransporter‐2 inhibitors, metformin), it would seem logical that these drugs would work well in particular patient subgroups. Insulin secretagogues require some preserved β‐cell function to work. A recent study on glucagon‐like peptide‐1 receptor agonists showed that patients with Type 2 diabetes with severe insulin deficiency (fasting C‐peptide < 0.25 nmol/l) had markedly reduced glycaemic response, with an HbA_1c_ reduction of only 5.2 mmol/mol in this group compared with 15.2 mmol/mol in those with preserved β‐cell function 4. Conversely, thiazolidinediones have been reported to work more effectively in obese insulin‐resistant patients compared with patients of normal weight 5. There are surprisingly few studies that have comprehensively assessed fasting C‐peptide or other measures of insulin secretion and sensitivity in relation to response to diabetes therapies, and this would seem a likely fruitful area for further study, as C‐peptide is a simple‐to‐measure biomarker that is a useful marker of underlying disease pathophysiology.

## Monogenic aetiology

Aetiologically there has been a tendency to treat all Type 2 diabetes as one overarching entity, yet we now know that it is possible to dissect the aetiology of Type 2 diabetes, especially when considering potential monogenic forms of diabetes such as maturity‐onset diabetes of the young (MODY) and familial partial lipodystrophy. MODY caused by mutations in the HNF1A gene is a very good example of how dissecting the aetiology of diabetes leads to personalized treatment. After case reports of sulphonylurea sensitivity in this patient group, a randomized crossover trial of sulphonylureas and metformin in patients with HNF1A MODY and patients with Type 2 diabetes established that patients with this subtype of MODY are exquisitely sensitive to sulphonylurea treatment 6. This most likely relates to the fact that the defects in the β cell caused by HNF1A mutations are in glycolysis and mitochondrial metabolism, and are therefore largely bypassed by sulphonylurea treatment, which acts downstream on the K_ATP_ channel. This work has resulted in the successful transition off insulin treatment and improved patient care for this subgroup of patients 7; however, this success highlights another challenge of personalized care: implementation. It is now more than 10 years since this result was published, yet some areas of the UK have very low referral rates for molecular genetic testing in diabetes 8 which must result in many patients being inappropriately treated. A more systematic approach to detection of monogenic disease is required.

## Drug disposition

For a drug to be effective it has to reach its site of action at a sufficient concentration to elicit an effect. Pharmacogenetics has long focused on potential for variation in genes involved in drug transport and metabolism to alter drug concentrations and subsequently to alter drug action and side effects. For diabetes drugs the two most robust findings relate to the effect of variation in cytochrome P450 2C9 (*CYP2C9*) and sulphonylurea efficacy and the recent discovery that variation in organic cation transporter 1 (OCT1) alters tolerance to metformin.

Sulphonylureas are primarily inactivated in the liver by the cytochrome P450 2C9 enzyme. Whilst most people have a normal version of this enzyme, some carry reduced‐function polymorphisms in the gene encoding this enzyme, termed *2 and *3. In all, 6% of the population carry two reduced‐function polymorphisms and, as such, would be predicted to inactivate sulphonylureas poorly. A GoDARTS study from Tayside, UK, established that this 6% of the population with loss of function of CYP2C9 are 3.44 times more likely to achieve an HbA_1c_ target < 53 mmol/mol (7%) 9; however, as might be expected, increased drug concentrations as a result of poor sulphonylurea metabolism have also been associated with increased risk of hypoglycaemia, albeit in limited small studies 10, 11. It seems likely that these patients would benefit from a personalized approach to therapy, with lower starting doses of sulphonylurea. A genotype‐driven clinical trial is required to establish this before it can be implemented into clinical care.

**Figure 2:**
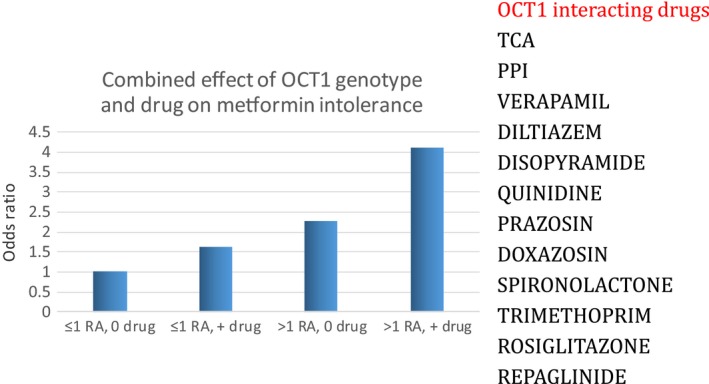
Odds ratio for severe metformin intolerance. RA, reduced‐function allele in organic cation transporter 1 (OCT1). 0 or + drug refers to the absence or presence of any potential OCT1‐interacting drug shown in the list.

Metformin, is an organic cation, and hence its disposition is largely influenced by the group of transporters called the organic cation transporters. Most focus has been on the role of genetic variation in OCT1 on metformin efficacy because OCT1 has an established role in metformin uptake into the liver 12; however, there is little consensus on the impact of this transporter on metformin response. OCT1 also has a role in metformin transport across the intestinal wall and it was hypothesized that it may play a role in metformin intolerance. We have recently established that the 8% of white Europeans who carry two reduced‐function variants in OCT1 are nearly twice as likely to develop severe metformin intolerance as those who have normal function in OCT1 13. This finding has subsequently been replicated in a small cohort with self reported mild metformin intolerance 14. Interestingly, we also showed that co‐prescribed drugs increase risk of intolerance 13. There are a number of drugs (see list in Fig. 2) that inhibit OCT1 transport, and whilst these have a small effect in their own right, the impact of these drugs on metformin intolerance is much greater in those who carry two reduced function OCT1 variants, with this group having a fourfold greater risk of gastrointestinal intolerance to metformin (Fig. 2). This means that patients with metformin intolerance who are treated with an OCT1‐interacting drug should be trialed on an alternative drug if possible, the most common of these drugs being the proton pump inhibitors; in such patients a trial of H_2_ receptor antagonists should be considered. If these results can be validated in a clinical trial, then it may be possible to consider a scenario where a lower metformin dose or a slow‐release preparation is used and co‐prescribed medication altered in the 8% of patients who carry the risk genotype.

## Insights from genome‐wide studies

The widespread introduction of low‐cost genome‐wide arrays has enabled the move from the study of single candidate genes, to the study of common variants across the whole genome. This approach has particular utility when the mechanism of action of a drug is uncertain, and hence a candidate gene approach is difficult. Despite the widespread use of genome‐wide association studies (GWAS) for most traits and common diseases, the application of GWAS to drug response has been limited. Probably the best example of GWAS applied to drug outcomes in conditions other than diabetes was the finding that variants in *SLCO1B1* (encoding the statin transporter OATP1B1) increase the risk of statin‐associated myopathy, with 2% of the population who carried two c‐alleles at rs4140956 being 16 times more likely to develop severe myopathy with simvastatin. The effect size meant that only 85 cases needed to be included and 90 controls.

The only reported GWAS for diabetes drug response has been for metformin. As the mechanism of action remains much debated, the hypothesis‐free approach of a GWAS offered considerable potential to gain insight into the molecular mechanism of action of metformin. The GoDARTS and UK Prospective Diabetes Study (UKPDS) metformin pharmacogenetics study group carried out a GWAS in ~1100 patients treated with metformin 15. In that study, one locus on chromosome 11 was associated with metformin response, with a *P* value = 1.9*10^−7^. This locus was subsequently replicated in two independent cohorts, including the UKPDS, with a combined overall *P* value = 2.9*10^‐9^. This genetic association has been subsequently replicated in additional European cohorts 16 and a Chinese cohort 17, making this the most robust metformin pharmacogenetic variant for metformin efficacy to date. The locus on chromosome 11, tagged by rs11212617, consists of a large LD block encompassing seven genes. There is considerable supporting literature to point to the *ATM* gene as the likely candidate at this locus. *ATM* encodes a DNA damage protein that is faulty in some cancers. Somatic recessive mutations in *ATM* cause ataxia telangiectasia, a syndrome characterized in part by increased risk of cancer and diabetes 18, 19. We have recently confirmed that patients with ataxia telangiectasia have impaired glycaemia and insulin resistance, which supports the hypothesis that ATM plays a key role in insulin metabolism 20. The exact mechanism whereby variation in *ATM* or its adjacent partner gene *NPAT*, alters metformin response is a focus of ongoing work.

## Beyond genomics

To date personalized medicine in diabetes, and indeed in most diseases, has focused on DNA sequence variation; however, this only captures a fraction of the overall complexity of human variation. As technology continues to drive forward, we are now moving into a field that is far more complex, that takes into account tissue‐specific epigenetics (epigenomics) and gene expression (transcriptomics), and the integration of this expression data with environmental and drug exposures that can be captured on large‐scale targeted and non‐targeted assays of metabolites (metabolomics) and proteins (proteomics). There is also an increasing recognition of the role the gut microbiome plays in metabolism, and in particular drug metabolism 21; genetic sequencing approaches are now increasingly used to identify the bacterial species present in the gut and relate this to disease risk or drug exposure. These approaches have not yet been applied to the study of drug outcome in diabetes but have been reported for other drugs and outcomes. For example, in the field of pharmacometabolomics (reviewed in Kaddurah‐Daouk *et al*. 22), the metabotype has been shown to alter treatment response to selective serotonin re‐uptake inhibitors 23. The gut microbiome has been known to affect drug disposition for many years. For example, 10% of the population are colonized with the intestinal anaerobic bacterium *Eubacterium lentum*, which metabolizes and inactivates > 40% of ingested digoxin before it is absorbed 24; the co‐adminstration of antibiotics that disrupt this inactivation results in cardiotoxicity 25.

Intriguingly, metformin is recognized as playing an increasing role in the gut 26 and has been recently shown to alter the microbiome in a way that may account for at least some of the intolerance and efficacy associated with metformin treatment 27. The study of the microbiome, and associated host metabolome, in relation to metformin response is, therefore, likely to be an area of increasing interest. How this will translate into personalized therapy will be interesting, but some studies have already assessed the impact of a microbiome modulator on metformin intolerance with some success 28.

## Conclusions

All clinicians aim to practise personalized medicine, but to date we are not armed with sufficient evidence to truly personalize treatment, resulting in the need for an educated guess or a trial‐and‐error approach. The modern era of personalized medicine is moving towards identifying clinical and molecular signatures than predict a therapeutic outcome, reducing the uncertainty in treatment decisions, i.e. making treatment more precise. We are however at the beginning of this process, with only few robust examples of phenotype or genotype guiding treatment choice. The recent technological advances enable a much greater understanding of individual variability that may alter outcome, but also vastly increase the complexity of studies aiming to identify such predictive biomarkers. It seems highly likely that the next 10 years will deliver major advances in personalized medicine in diabetes; what seems even more likely is that it will not be called personalized or even precision medicine by then.
